# An Uncommon Case of Renal Metastasis from Cervical Cancer

**DOI:** 10.7759/cureus.1941

**Published:** 2017-12-13

**Authors:** Amine Bazine, Hanae O Zniber, Meriem Ghaouti, Aziz Bazine, Aziz Baydada, Hassan SIFAT

**Affiliations:** 1 Department of Radiotherapy, Military Hospital Mohamed V, Rabat, Morocco; 2 Department of Gynecology-Obstetrics M1, Ibn Sina University Hospital Center, Rabat, Morocco; 3 Nations Unies Pathology Center; 4 Department of Medical Oncology, Military Hospital Moulay Ismaïl, Meknès, Morocco; 5 Department of Gynecology-Obstetrics, Ibn Sina University Hospital Center, Rabat, Morocco

**Keywords:** renal metastasis, cervical cancer, relapse

## Abstract

Metastases to the kidney are a rare entity. Among solid tumors, it is known that lung and colorectal cancers can metastasize to the kidney. Renal metastases from cervical cancer are exceptional; only 12 cases were previously reported. We report a case of a right renal metastasis from a cervical squamous cell carcinoma, occurring in the context of a metastatic relapse two years after completing primary treatment.

## Introduction

Cervical cancer continues to be a significant health problem worldwide. Although uncommon at initial diagnosis, metastatic disease will develop in 15% to 61 % of women with cervical cancer, usually within the first two years of completing treatment [1]. The occurrence of metastases is associated with poor outcomes. The most frequent metastatic sites include lung, lumbar and thoracic spine, and para‑aortic lymph nodes.

Renal metastases are extremely rare with only 12 cases previously reported. Practically, the main challenge is to link renal metastasis to primary cervical cancer to rule out the diagnosis of primary renal carcinoma. Histological and immunohistochemical analyses of the renal tumor, communicated by biopsy or nephrectomy, are a very important step for this purpose.

We report here a rare case of right renal metastasis from primary cervical cancer, occurring two years after concurrent chemoradiotherapy, and discovered in the context of a diffuse metastatic relapse.

## Case presentation

A 47-year-old female was followed since May 2014 for stage IIb International Federation of Gynecology and Obstetrics (FIGO) cervical squamous cell carcinoma without positive pelvic or para-aortic lymph nodes on magnetic resonance imaging (MRI) and computed tomography (CT) performed to evaluate loco-regional extension. Positron emission tomography-computed tomography (PET-CT) or para-aortic lymph node sampling to evaluate para-aortic lymph node status was not initially performed. She received concomitant chemoradiation (pelvic radiotherapy consisting of 46 Gy in 23 fractions of two Gy/fraction; chemotherapy consisting of cisplatin at a dose of 40 mg/m²/week for five weeks) followed by uterovaginal brachytherapy (four fractions of seven Gy). For two years after completion of treatment, the patient did not report any alarming symptoms. Physical examination and imaging studies of the patient showed no signs of recurrence.

**Figure 1:**
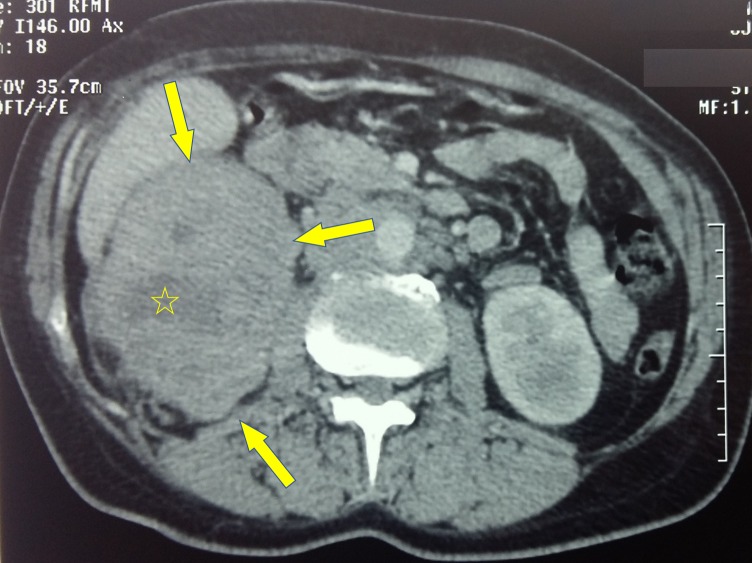
Right renal mass Axial CT scan showing large right renal mass (denoted by arrows), poorly defined, heterogeneous, and containing hypodense regions of tumor necrosis (denoted by the star). CT, computed tomography.

In December 2016, the patient started having a dry cough, costal and spinal pain, and weight loss. She did not report metrorrhagia or vaginal discharge. A whole-body CT scan showed a poorly defined, heterogeneous, large, right renal mass that captured contrast discreetly and contained hypodense regions of tumor necrosis (Figure 1).

**Figure 2:**
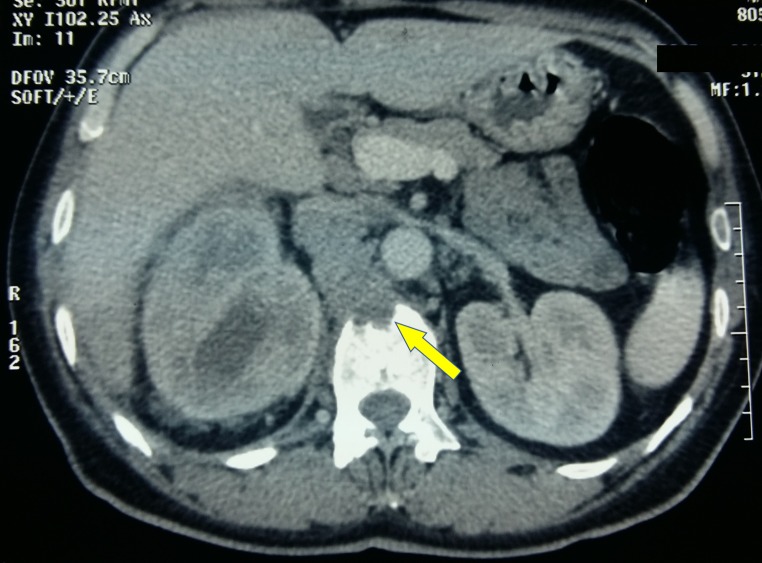
Para-aortic lymph node mass Axial CT scan showing a para-aortic lymph node mass that invaded the L1 vertebral body. CT, computed tomography.

Additionally, the scan found a para-aortic lymph node mass that encompassed the right renal artery and invaded the L1 vertebral body (Figure 2).

The CT scan also noted multiple metastases in the lungs and bone. A pelvic MRI showed no signs of recurrence in the pelvis.

**Figure 3:**
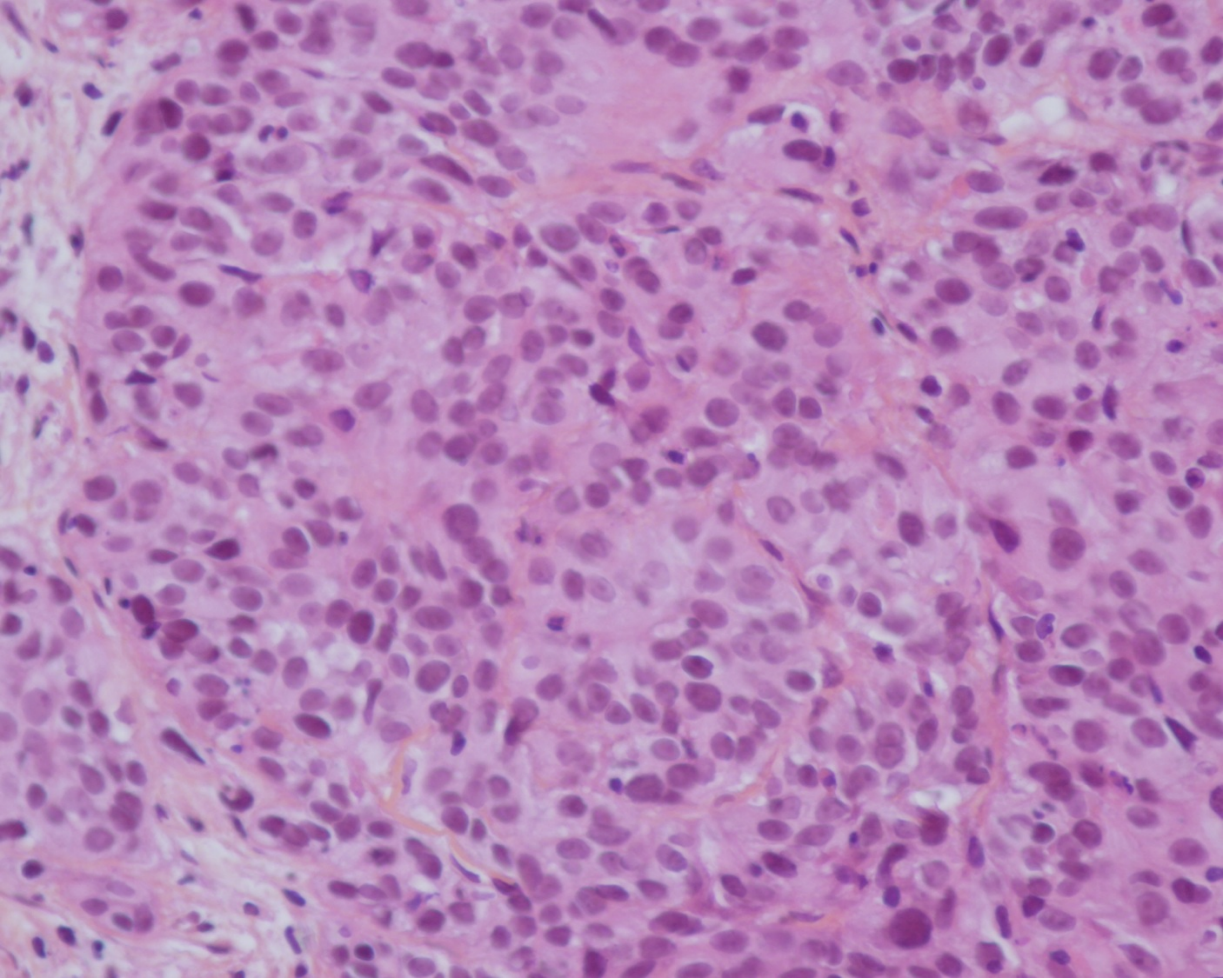
Squamous cell carcinoma Pathological examination revealing squamous cell carcinoma that was similar to the pattern of the original cervical carcinoma (hematoxylin and eosin staining; magnification, x400).

Two diagnoses had been suggested. The first was a metastatic recurrence of the squamous cell carcinoma of the cervix and the second was metastatic renal carcinoma. Biopsies of the right renal mass and pulmonary metastases were performed via a percutaneous route. A pathological analysis of the biopsies showed that the tumor proliferation was composed of squamous cells similar to the pattern of the original cervical carcinoma (Figure 3).

**Figure 4:**
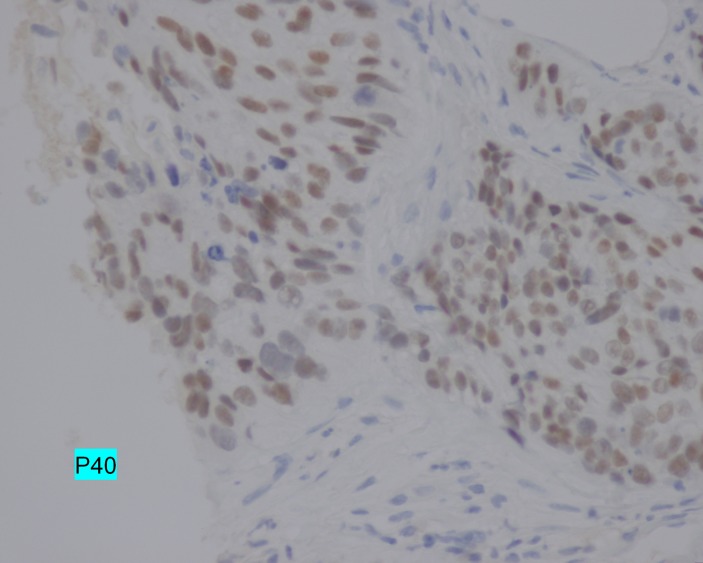
p40 expression Immunohistochemical analysis demonstrating the positive nuclear expression of p40 (magnification, x400).

**Figure 5:**
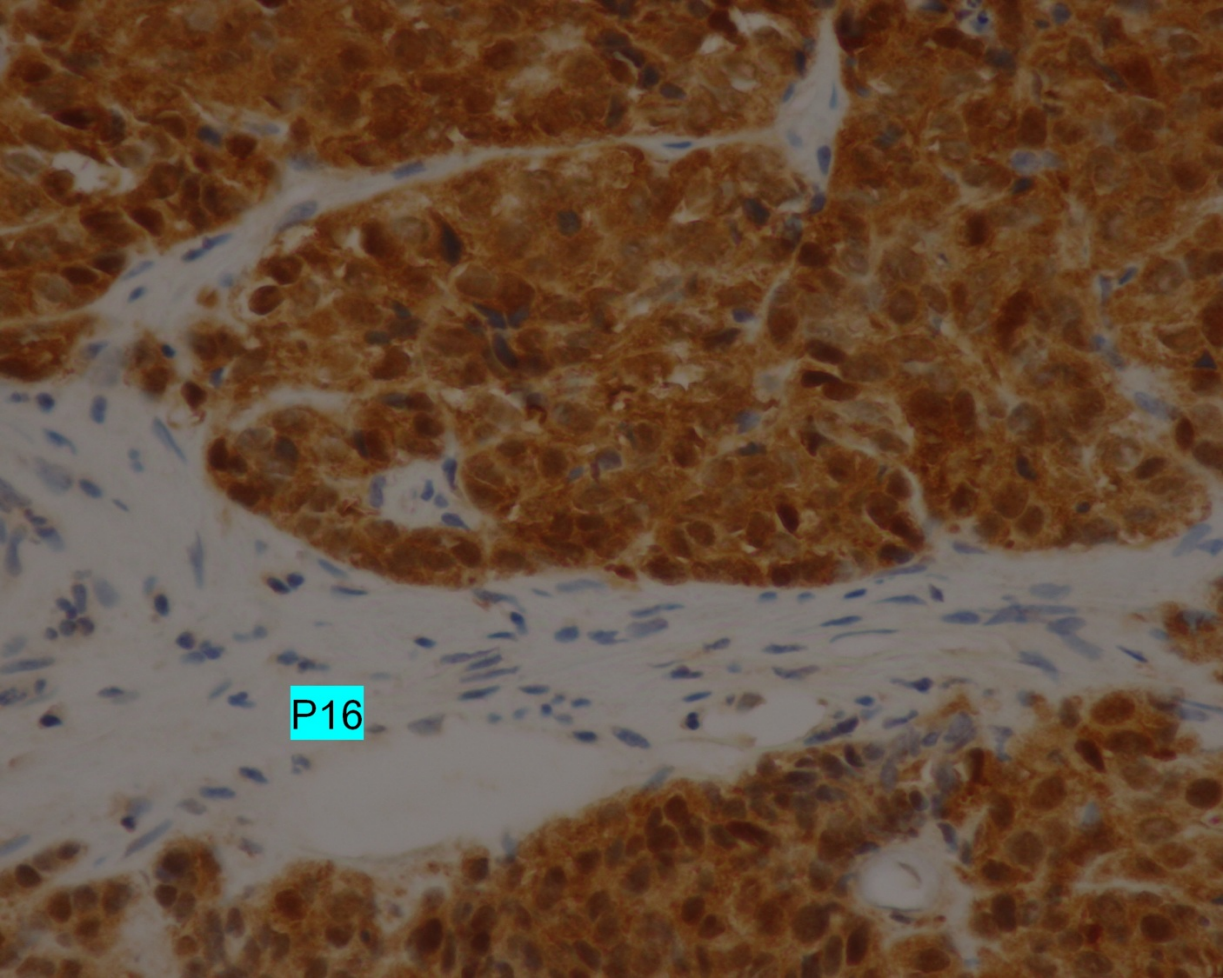
p16 overexpression Immunohistochemical analysis demonstrating the strong and diffuse positive nuclear and cytoplasmic expression of p16 (magnification, x400).

An immunohistochemical analysis of the biopsies indicated that the tumor was highly positive for p40 (Figure 4) and p16 (Figure 5).

Consequently, the pathological and immunohistochemical examination confirmed a diagnosis of metastatic squamous cell carcinoma, histologically consistent with the original cervical carcinoma experienced by the patient two years prior.

Once the diagnosis of metastatic relapse was made, palliative chemotherapy based on 5-fluorouracil and carboplatin was started. The patient died three months after the diagnosis of relapse.

## Discussion

Most of the literature on renal metastases comes from autopsy data. Bracken et al. reviewed 11,328 autopsies performed on patients who died of malignant disease; 7.2% (816 cases) demonstrated renal metastases [2]. This appears to be even more frequent than primitive renal carcinoma. However, clinicopathological detection of renal metastases is more infrequent. The largest and most comprehensive study of this clinical scenario to date comes from the University of Texas MD Anderson Cancer Center. It reports, over a period of about 30 years, only 151 cases of renal metastases. The most common primary tumor sites in this study were lung (43.7%), colorectal (10.6%), head and neck (6%), breast (5.3%), soft tissue (5.3%), and thyroid (5.3%) [3].

If all renal metastases are rare, those of cervical origin are even more so. In an autopsy series, cervical cancer represented only 2.5% of cancer metastasis to the kidney [4]. In the MD Anderson Cancer Center study, no case of renal metastasis of cervical origin was reported [3]. To our knowledge, only 12 cases have been reported to originate from cervical carcinoma to date; they are all reported as case reports [5-9].

The circumstances of discovery, reported in the literature, are very variable. Renal metastases often cause symptoms that may simulate a renal-abscess-like flank pain, haematuria, or fever [5]. Renal metastases can also be discovered during follow-up imaging studies [5,8]. In the present case, the renal metastasis was found during a whole-body CT performed for symptoms related to lung and bone metastases (cough and bone pain).

In the majority of cases, renal metastases occur in the context of a metastatic relapse after a free interval from diagnosis and primary treatment, with the longest period lasting 118 months [5]. In one case, renal metastasis was indicative of cervical cancer [9]. In our case, metastatic relapse occurred 31 months after the initial diagnosis. It was diffuse, affecting lungs, bones, and the right kidney, with a para-aortic lymph node mass. This could suggest that there was a para-aortic involvement, unnoticed in the initial workup, which was at the origin of the metastatic relapse. That is why it is recommended to perform a PET-CT at diagnosis, from stage IB2 FIGO.

Histological evidence can be provided by biopsy or nephrectomy. In this context, overexpression of the p16 protein is a useful marker for confirming the cervical origin of renal metastasis. The p16 protein is present in the basal cells of the squamous epithelium of the cervix, as a result of the expression of the viral oncogene E7 during high-risk human papillomavirus (HPV) infection associated with 95% of cases with invasive cervical cancer [10]. In our patient, p16 overexpression was objectified by immunohistochemistry on both the right renal mass and the pulmonary metastases.

There is no standard treatment for patients with metastatic cervical cancer because of its heterogeneous manifestations. In case of renal metastases, nephrectomy should be preferred if the metastatic lesion is located unilaterally in the kidney and if the patients' performance status is good. However, this treatment may only relieve the clinical symptoms of the patient [8]. Currently, outcomes of patients with metastatic cervical cancer are poor. In the present case study, the patient died only three months after the diagnosis of relapse was confirmed, testifying to a very aggressive disease.

## Conclusions

Renal metastases from cervical cancer are exceptionally rare. During the follow-up of patients treated for cervical cancer, the discovery of a nodule or renal mass should lead to a biopsy (or nephrectomy, if the lesion is unique) to confirm the diagnosis of metastasis from cervical cancer. For this purpose, research of p16 overexpression status via immunohistochemistry is useful. Patients with metastatic cervical cancer, including those with renal metastasis, have poor outcomes.
